# Does NGAL reduce costs? A cost analysis of urine NGAL (uNGAL) & serum creatinine (sCr) for acute kidney injury (AKI) diagnosis

**DOI:** 10.1371/journal.pone.0178091

**Published:** 2017-05-19

**Authors:** Amay Parikh, John A. Rizzo, Pietro Canetta, Catherine Forster, Meghan Sise, Omar Maarouf, Eugenia Singer, Antje Elger, Saban Elitok, Kai Schmidt-Ott, Jonathon Barasch, Thomas L. Nickolas

**Affiliations:** 1 Columbia University Medical Center, New York, New York, United States of America; 2 Departments of Preventive Medicine & Economics, Stony Brook University, Stony Brook, New York, United States of America; 3 Staten Island University Hospital, Staten Island, New York, United States of America; 4 Charité - Universitätsmedizin Berlin, Helios Clinics Berlin, Max-Delbrück-Center for Molecular Medicine, Berlin, Germany; The University of Tokyo, JAPAN

## Abstract

**Introduction:**

Urine neutrophil gelatinase-associated lipocalin (uNGAL) is a sensitive and specific diagnostic test for acute kidney injury (AKI) in the Emergency Department (ED), but its economic impact has not been investigated. We hypothesized that uNGAL used in combination with serum creatinine (sCr) would reduce costs in the management of AKI in patients presenting to the ED in comparison to using sCr alone.

**Materials and methods:**

A cost simulation model was developed for clinical algorithms to diagnose AKI based on sCr alone vs. uNGAL plus sCr (uNGAL+sCr). A cost minimization analysis was performed to determine total expected costs for patients with AKI. uNGAL test characteristics were validated with eight-hundred forty-nine patients with sCr ≥1.5 from a completed study of 1635 patients recruited from EDs at two U.S. hospitals from 2007–8. Biomarker test, AKI work-up, and diagnostic imaging costs were incorporated.

**Results:**

For a hypothetical cohort of 10,000 patients, the model predicted that the expected costs were $900 per patient (pp) in the sCr arm and $950 in the uNGAL+sCr arm. uNGAL+sCr resulted in 1,578 fewer patients with delayed diagnosis and treatment than sCr alone (2,013 vs. 436 pts) at center 1 and 1,973 fewer patients with delayed diagnosis and treatment than sCr alone at center 2 (2,227 vs. 254 patients). Although initial evaluation costs at each center were $50 pp higher in with uNGAL+sCr, total costs declined by $408 pp at Center 1 and by $522 pp at Center 2 due to expected reduced delays in diagnosis and treatment. Sensitivity analyses confirmed savings with uNGAL + sCr for a range of cost inputs.

**Discussion:**

Using uNGAL with sCr as a clinical diagnostic test for AKI may improve patient management and reduce expected costs. Any cost savings would likely result from avoiding delays in diagnosis and treatment and from avoidance of unnecessary testing in patients given a false positive AKI diagnosis by use of sCr alone.

## Introduction

AKI has been associated with significantly increased health care costs. Data from 23 Massachusetts hospitals over 2 years demonstrated that AKI resulted in higher hospital resource utilization, as both median direct hospital costs and hospital length of stay were increased by $2,600 and by 5 days, respectively, in patients with AKI vs. patients without AKI.[1] Furthermore, Chertow et al. demonstrated that even uncomplicated AKI was associated both with greater hospital costs and longer lengths of stay.[2] In the current and future eras of limited healthcare budgets, it is critically relevant to evaluate the potential costs and economic impact of novel diagnostic tests.

Measurement of urinary neutrophil gelatinase-associated lipocalin (uNGAL) has been demonstrated to be an early, non-invasive marker of AKI due to a variety of etiologies, such as cardiac surgery[3], intravenous contrast administration[4], critical care settings[5], and kidney transplantation[6]. NGAL is the most studied marker of AKI and was demonstrated to be sensitive and specific in the detection of AKI in the ED and for the prediction of a composite clinical outcome of death and dialysis after in-patient admission [7, 8]. It is the most prominently studied because i) it has an enormous dynamic range ii) responds in a dose-dependent fashion to injury iii) responds within 3 hours of injury, which is important in emergency rooms iv) responds to a wide range of injuries v) is easy to measure vi) due to the recent availability of clinical platforms including a new NGAL dipstick vii) the test has been approved in Japan, Korea, and parts of Europe. It is currently being reviewed by the FDA in the US. In comparing a variety of potential biomarkers for study, the authors found NGAL tracks with significant changes in levels of creatinine, and had a better AUC/ROC.[9] Thus, uNGAL values may then be used to initiate AKI patient care algorithms earlier than sCr alone, and the application of uNGAL to AKI diagnostics and management may potentially lead to improved patient outcomes.

Although multiple investigations have demonstrated that uNGAL is a promising AKI biomarker with a potential application in the ED and other acute settings, little data exist regarding its potential impact upon costs and resource utilization. Therefore it is at this time that a cost analysis is particularly valuable at this time. Even though patients present to the ED with a variety of conditions, which may or may not include AKI, our study chose to focus exclusively on creatinine, the AKI diagnosis, and its cost implications. We therefore investigated the economic impact of uNGAL measurement on AKI detection and management in patients presenting to the ED with sCr levels ≥ 1.5 mg/dL. From a diagnostic perspective this level of sCr is relatively non-specific as it may represent prerenal azotemia or stable CKD, in addition to AKI. Moreover, a sCr above this cutoff, in the absence of baseline kidney function data, would likely trigger clinicians to initiate an AKI clinical care pathway. We hypothesized that early and accurate AKI detection by uNGAL, in conjunction with sCr, would lead to more efficient and less expensive patient care.

With limited clinical and economic data, the relationship between cost and efficacy of an additional diagnostic biomarker becomes even more important for EDs when considering adopting such a test. Given the lack of complete and perfect information, economic modeling techniques are employed to determine cost-effectiveness. Such a model allows one to synthesize data from a variety of sources and estimate the clinical and cost outcomes on different populations. Sensitivity analysis of the model allows one to verify the assumptions made in the model and test the validity of uncertain data (including costs) usually obtained from a variety of sources. Knowing the limitations of such modeling, we constructed a decision model to compare the effectiveness of the addition of uNGAL to sCr versus sCr alone.

## Materials and methods

A cost simulation model was developed (Microsoft Excel, Redmond, WA) for competing AKI diagnostic strategies; 1) sCr alone; and. 2) uNGAL plus sCr (uNGAL+sCr). The model was used to simulate a cohort of 10,000 adult patients with creatinine greater than or equal to 1.5 mg/dL from the period of presentation to the emergency department to hospital admission at each hospital site in the United States. Costs were examined from the payer perspective.

Data for the model was obtained from the published literature. Additionally rates for outcomes were obtained from a previous randomized controlled trial.[9] This dataset derived from a broad and unbiased multicenter trial including a variety of races, locations, age, gender, and socioeconomic statuses.[9] All comers consecutively to emergency rooms were included. Inclusion criteria were broad and exclusion criteria were limited to appropriate groups for consent purposes. ESRD was excluded as the authors wished to follow the reversibility of AKI. Our present analysis derives from the review of this dataset. This study was approved by the Institutional Review Board of Columbia University Medical Center and Staten Island University Hospital, and performed in compliance with HIPPA. Written, informed consent was obtained from all participants.

### Model structure

**Fig 1:**
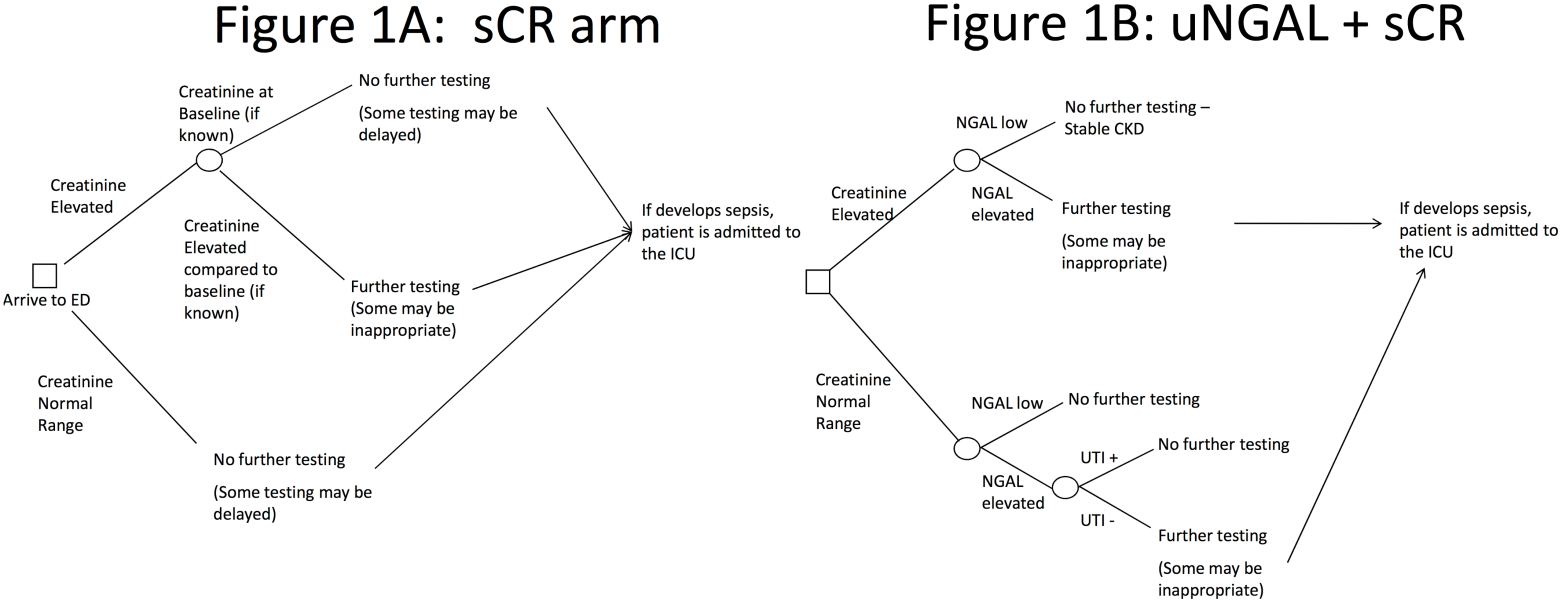
Simulation model. A cost simulation model was developed for competing testing strategies to evaluate for AKI; 1) Scr alone, vs. 2) uNGAL plus Scr (uNGAL+Scr). Since the uNGAL+Scr treatment arm provided more diagnostic information, it was regarded as the “gold standard” relative to Scr alone in terms of whether patients should be treated or whether treatment should be delayed.

On the basis of the recommended clinical management of patients presenting to the ED with AKI, the model was composed of two alternatives: sCr alone (Fig 1A) and sCr + NGAL (Fig 1B). This decision-tree was constructed based on previous literature [10, 11] and qualitative analysis with expert opinion. The decision-tree framework provided the clinical testing pathways.

For evaluation of sCr alone, patients with a level below the cutoff were not evaluated further for kidney injury (Fig 1A). For uNGAL+sCr, four outcomes were possible (Fig 1B): (1) both tests above cutoff levels; (2) sCr above cutoff level but uNGAL below cutoff level; (3) sCr below cutoff level but uNGAL above cutoff level; and (4) both tests below cutoff levels. In case 1, all patients were referred for further AKI testing. In case 4, no patients were referred for further AKI testing. In case 2, patients were referred for further AKI testing unless they had known stable chronic kidney disease (CKD) and in case 3, patients were referred for further AKI testing unless they had evidence of urinary tract infection (UTI).

### Input parameters

Data collected at NYP-Allen and SIUH provided the prevalence of both elevated sCr and uNGAL, of UTIs, SIRS, and sepsis. For the baseline case, an elevated sCr level was assigned a value ≥1.75 mg/dL and an elevated uNGAL level was assigned a value ≥ 125 ng/dL. In both the sCr alone and uNGAL+sCr groups, a hypothetical cohort of 10,000 patients with suspected AKI were evaluated and “run through” the model. Based on adjudication results of the study completed at each site, we determined the prevalence of each condition. The prevalences then allowed the calculations for the sensitivity and specificity in detecting AKI for sCr and uNGAL.

### Model assumptions

There are three sets of assumptions inherent to this analysis. The first set of assumptions is dependent upon the false negative-AKI rate: (1) Patients in the ED with a sCr level below the cutoff are sent home; (2) This results in a number of false negative-AKI diagnoses and a delay in management; and (3) Those patients with a false-negative diagnosis based on sCr alone will return to the ED, with a more severe disease state.

The second set of assumptions is dependent on the false positive-AKI rate: (1) Patients assigned a false-positive AKI diagnosis were admitted to the hospital for an elevated sCr; (2) A percentage of these hospital admissions were unnecessary (i.e. stable CKD, they could have been discharged); and (3) Unnecessary testing was performed.

The third set of assumptions is dependent on the treating physician’s clinical approach: (1) All patients in the ED with sCr above the cutoff level received AKI diagnostic testing; (2) Additional testing was obtained in those patients admitted to the hospital. Clinical criteria and results of these tests determined whether the patient had the systemic inflammatory response syndrome (SIRS) or sepsis, and whether they would be admitted to the general wards or the intensive care unit (ICU).

### Patient population

Between January 1, 2007 and December 31, 2008, 1,120 consecutive patients were recruited from the EDs at Staten Island University Hospital (SIUH) and the Allen Hospital of the New York-Presbyterian Hospital (AH-NYPH) as part of a multicenter clinical study, which is detailed elsewhere.[12] Data regarding sCr values, clinical history and diagnostic tests were collected. Due to poor specificity for discrimination of AKI, prerenal azotemia and stable CKD at high levels of sCr, we restricted the cohort to only those patients having sCr levels ≥ 1.5 mg/dL and from this used a sCr value of ≥ 1.75 as elevated. We hypothesized that above this threshold, an AKI patient care algorithm, including serial sCr testing and renal ultrasound, may likely be required to accurately discriminate AKI from other forms of kidney dysfunction and that uNGAL testing could potentially provide useful diagnostic information regarding kidney function for this set patients. This restriction left a sample of 476 patients as SIUH and 373 patients at AH-NYPH.

Patients ≥18 years old in the ED, without a history of end stage renal disease (ESRD) and without a need for renal replacement therapy, who were being admitted to the hospital were enrolled from the ED at each site. One urine sample was collected and patient medical records were accessed. Study team members were not in contact with treating physicians, except to obtain permission to enroll patients.

### Clinical efficacy and model outcomes

The primary clinical effect of the decision pathways was either hospital admission from the emergency department or discharge to home. The model incorporated other clinical events such as the recognition of a UTI, SIRS, or sepsis.

### Costs

Data on hospital unit costs (e.g., daily costs for an inpatient stay) were obtained from Medicare cost reports (Table 1). Baseline sCr cost was assumed to be $100 [13] with a range of $50-$200 if bundled with other tests. For uNGAL, baseline cost was $50, with a range of $25—$100 used in sensitivity analysis [personal communication, Abbott Laboratories]. Other costs included additional testing to identify AKI and possible complications (e.g. SIRS and sepsis).[14] Testing to identify AKI included renal ultrasound, urinalysis, urine culture, and complete blood cell count with differential. Additional testing to determine the presence of sepsis included blood cultures. Total hospital and ICU length of stay were obtained from patients’ medical records at each center. Hospital and ICU length of stay for patients with and without AKI were compared and used to derive an incremental length of stay due to kidney disease. An estimate for this incremental length of stay was varied throughout a wide range of values in the sensitivity analysis.

**Table 1:** Model input values for each site.

MODEL INPUT	ALLEN	SIUH	SOURCE FOR BASELINE VALUES
**Cohort Size**	10,000	10,000	N/A
**Scr Cost**	100 (50–200)	100 (50–200)	[13]
**uNGAL Cost**	50 (25–100)	50 (25–100)	Abbott Laboratories
**Cost of Further Testing**	800 (400–1,600)	800 (400–1,600)	[2, 13]
**Cost of Test for SIRS**	300 (150–600)	300 (150–600)	[14]
**Cost of Test for Sepsis**	100 (50–200)	100 (50–200)	[14]
**Daily Hospital Cost**	2,000 (1,000–4,000)	2,000 (1,000–4,000)	[15]
**Daily ICU Cost**	6,000 (4,000–8,000)	6,000 (4,000–8,000)	[15]
**Average Incremental LOS in Hospital for Kidney Injury**	2 (1–4)	2 (1–4)	Derived from Dataset
**Adjusted Average Incremental LOS**^a^	1 (0.5–2)	1 (0.5–2)	Derived from Dataset
**Average Incremental LOS in ICU for Kidney Injury**	3 (1.5–6.0)	3 (1.5–6.0)	Derived from Dataset
**Probability Scr Not Elevated**	0.35(0.35–0.73)	0.29 (0.00–0.37)	Dataset [9]
**Probability uNGAL Not Elevated**	0.63 (0.50–0.63)	0.62 (0.56–0.62)	Dataset [9]
**Percent True Positive Scr**	0.34 (0.36–1.00)^b^	0.30 (0.34–1.00)^b^	Dataset [9]
**Percent True Negative Scr**	0.86 (0.85–1.00)	0.93 (0.94–1.00)^b^	Dataset [9]
**Percent True Positive NGAL**	0.52 (0.46–0.52)	0.40 (0.38–0.40)	Dataset [9]
**Percent True Negative NGAL**	0.87 (0.85–0.88)	0.85 (0.85–0.86)	Dataset [9]
**Percent Patients with UTI**	0.19 (0.10–0.38)	0.14 (0.07–0.28)	Dataset [9]
**Percent Patients with Stable CKD**	0.22 (0.11–0.44)	0.38 (0.19–0.76)	Dataset [9]
**Percent Patients with SIRS**	0.32 (0.16–0.64)	0.31 (0.16–0.62)	Dataset [9]
**Percent Patients with SIRS who become septic**	0.06 (0.03–0.12)	0.01 (0.005–0.02)	Dataset [9]

Data on hospital unit costs were obtained from Medicare cost reports. Each cost component was varied from one-half to twice its baseline value. A range for elevated Scr and uNGAL values were also modeled in the sensitivity analysis: 1.5 to 2 mg/dL for Scr and 100 to 150 ng/mL for uNGAL.

^a^ Adjusted average incremental costs pertain to patients who were admitted inappropriately—we assume that LOS is shorter for them.

^b^ The baseline percent TP for Scr is based on the Scr> 1.75 criterion. For Scr> 1.5 and Scr >2 criteria, the percent TP for Scr were both greater than in the baseline case. Similarly, the percent TN for Scr at the SIUH site was less in the baseline case (Scr>1.75) than in either the Scr>1.5 or Scr >2 scenarios.

### Sensitivity analysis

Due to uncertainty in the value of model inputs and the range of values and costs likely to be captured in multiple different settings, sensitivity analyses were performed Table 1. Each cost component was varied from one-half to twice its baseline value. A range for elevated sCr and uNGAL values were also modeled in the sensitivity analysis: 1.5 to 2.0 for sCr and 100 to 150 for uNGAL. Ranges in the sensitivity analysis for the probability of elevated sCr and uNGAL tests were based on their associated cutoff value ranges.

## Results

### Baseline demographics

476 patients at SIUH and 373 patients at AH-NYPH were adjudicated and used to construct the model.

### Model results

**Fig 2:**
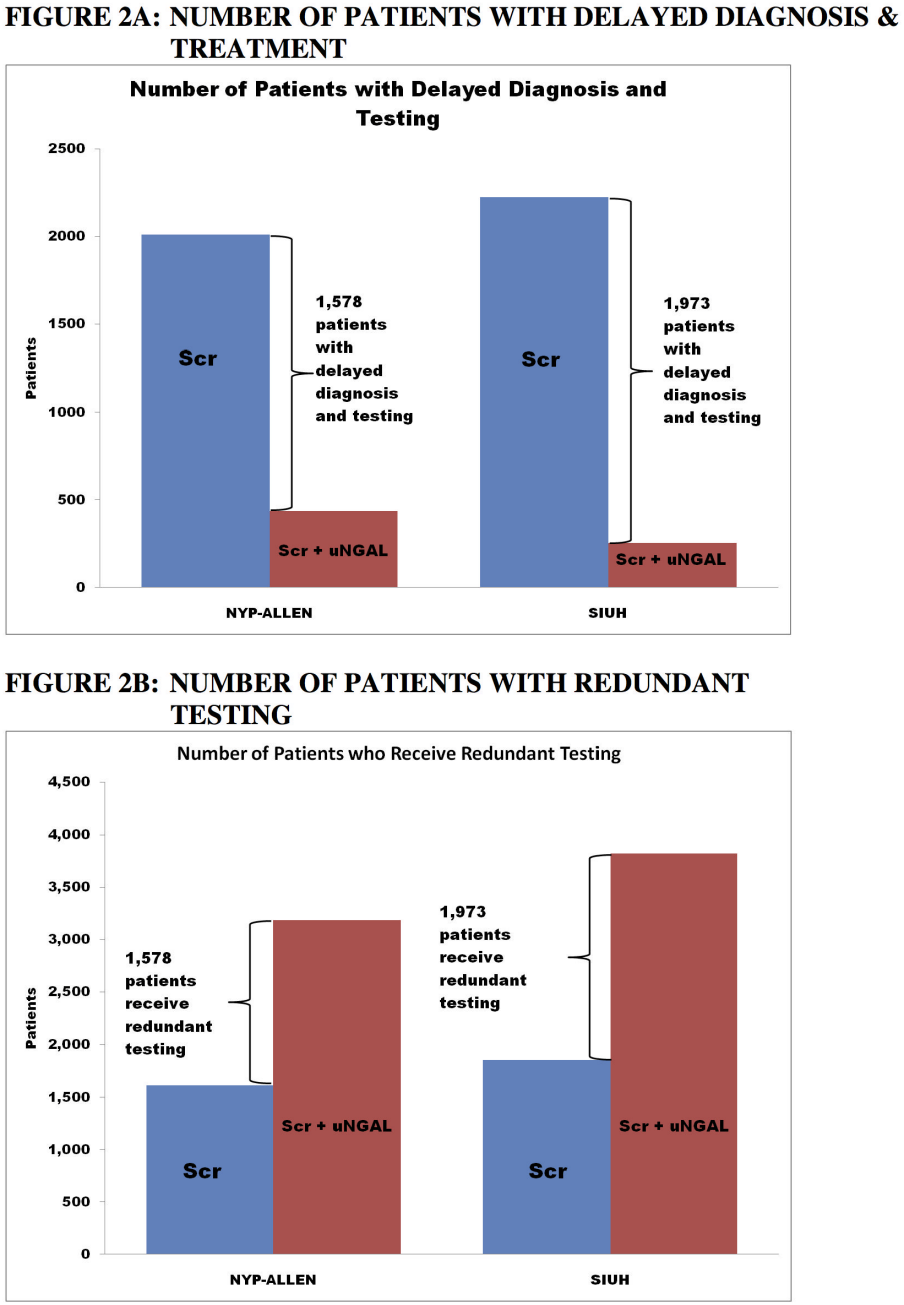
Delayed treatment and redundant testing. At both sites, the use of the Scr+uNGAL would be expected to reduce the number of patients with delayed diagnoses and treatment. For a hypothetical cohort of 10,000 patients, the Scr+uNGAL strategy would lead to 1,578 fewer patients with diagnosis and treatment delays at the NYP-Allen, and 1,973 fewer patients with such delays at SIUH. However, Scr+uNGAL could lead to redundant testing at both centers. …

The effects of sCr alone vs. uNGAL+sCr on expected treatment patterns are shown in Fig 2A and 2B. Fig 2A compares the two treatment groups in terms of the expected number of patients with delayed diagnosis and treatment. At both sites, the use of the uNGAL+sCr reduced the expected number of patients with delayed diagnoses and treatment. For a hypothetical cohort of 10,000 patients, using uNGAL+sCr would lead to 1,578 fewer patients with diagnostic and treatment delays at the AH-NYPH, and 1,973 fewer patients with such delays at SIUH. In contrast, Fig 2B reveals that uNGAL+sCr would also lead to more patients with potential redundant/unnecessary treatment at both centers.

**Fig 3:**
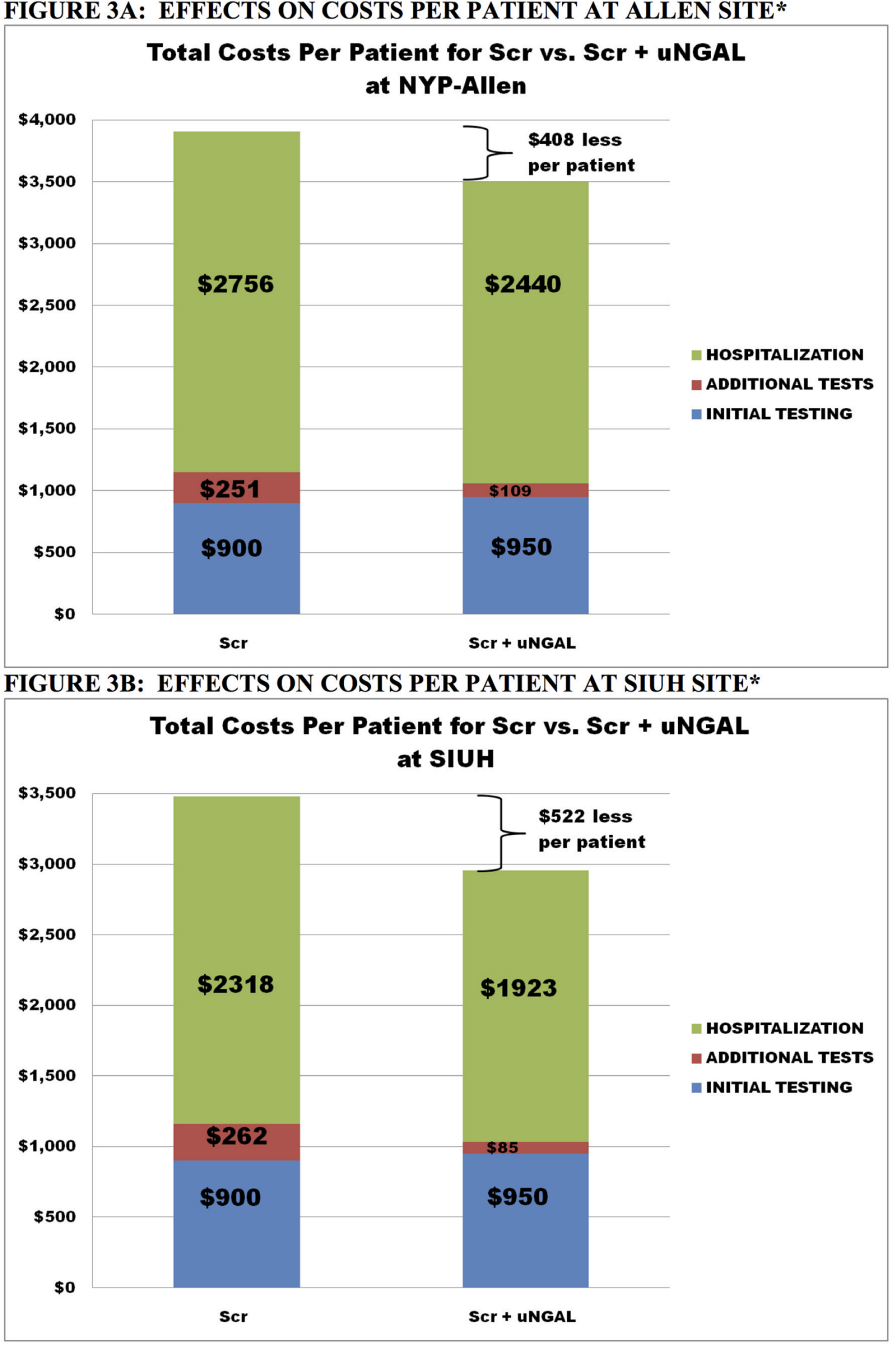
Effects on cost per patient. Effects on Cost Per Patient at NYP-Allen and SIUH. At NYP-Allen, the use of uNGAL+Scr would lead to an expected cost savings of $408 per patient on average, and to similar cost savings of $522 per patient at SIUH. These savings were reflected in lower per patient hospitalization costs and lower additional testing costs.

Fig 3A and 3B compare the two treatment arms in terms of cost per patient at the AH-NYPH and at SIUH, respectively. At AH-NYPH, the use of uNGAL+sCr would lead to an expected cost savings of $408 per patient on average, and to similar cost savings of $522 per patient at SIUH. These savings were realized as a result of lower expected per patient hospitalization costs (e.g. lower length of stay) and lower expected additional testing costs due to an early and appropriate diagnosis.

### Sensitivity analysis

**Fig 4:**
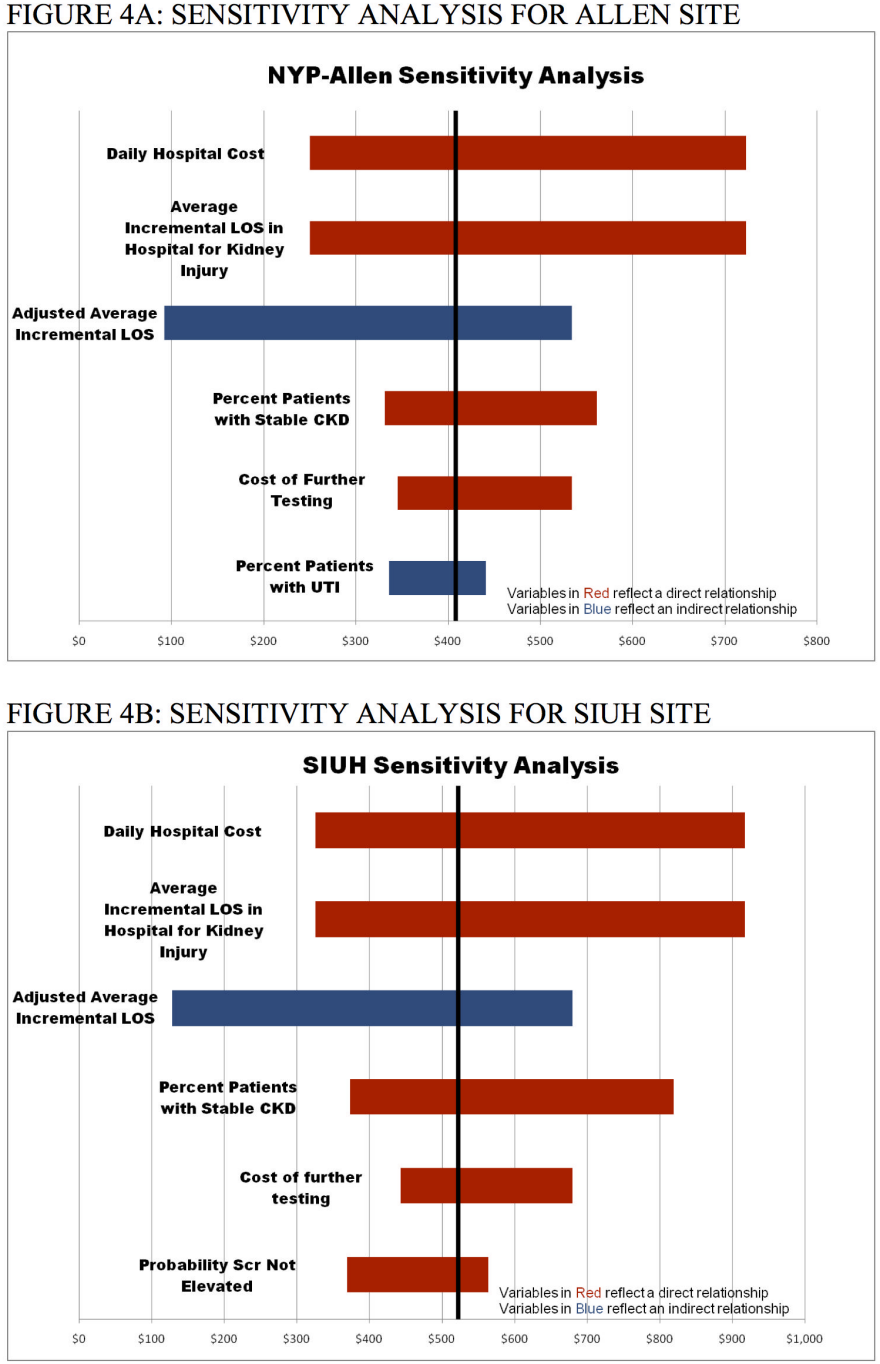
Sensitivity analysis. The sensitivity analysis recalculates the net expected cost of each strategy (Scr vs. uNGAL+Scr), varying one model input at a time to its high and low values relative to its baseline value. At both sites, costs of uNGAL+Scr remain lower for each scenario examined in the sensitivity analysis. At NYP-Allen and SIUH, the results are most sensitive to hospital costs and length of stay, costs of additional testing, and the percent of patients with CKD.

The results of sensitivity analysis are shown in Fig 4A for AH-NYPH and in Fig 4B for SIUH. The sensitivity analysis recalculates the net costs of sCr in comparison to uNGAL+sCr, varying one variable in the model at a time. The value of a given variable is adjusted to high and low values relative to its baseline value. At both sites, costs of uNGAL+sCr remained lower for each scenario examined in the sensitivity analysis. At AY-NYPH and SIUH, the results are most sensitive to hospital costs and length of stay, costs of additional testing, and the percent of patients with CKD. The results of the complete sensitivity analysis are available in the Appendix.

## Discussion

In the past 15 years, renal diagnosis has been simplified by the use of the term acute kidney injury (AKI). AKI severity can be stratified by either RIFLE, AKIN, or KDIGO criteria[16, 17]; higher AKI severity has been linked to poorer AKI-associated clinical outcomes.[18, 19] AKI refers to 2 criteria: rise in serum creatinine and decrease in urine output. The current definition as per KDIGO criteria is independent of etiology or type of injury. This change in nomenclature has the benefit of homogenizing the approach to the patient; however, it does not account for the fact of the different causes for the rise in creatinine have different treatments. Elevated sCr due to CKD, prerenal azotemia, or medication administration have no distinction from one another in KDIGO despite their drastically different clinical outcomes. Therefore, an AKI biomarker with improved specificity could improve both stratification of AKI severity and prediction of poor clinical outcomes. The NGAL gene and protein rise in the setting of ischemic and inflammatory damage to the kidney, but do not become elevated in volume depletion or related diseases like heart failure or liver failure. In the setting of AKI, uNGAL has been demonstrated to rise 24–48 hours before sCr and to more accurately differentiate AKI from prerenal azotemia.[20] Consequently, the fractionation of patients with elevated serum creatinine levels into 2 different diagnostic categories (NGAL+/Cr+, NGAL-/Cr+) demonstrates patients will respond to fluid therapy and those that will not.

Distinguishing various forms of AKI is a vexing problem. The time required for creatinine to reach a meaningful threshold is indeterminate: milligram quantities of creatinine must accumulate in the bloodstream from muscle. Consequently, the level of creatinine on presentation to the ED can never indicates the full extent of the defect in GFR for several days. sCr may vary based upon age, gender, muscle mass, nutritional status, medication usage, hydration status and existing co-morbid conditions.[21] For example, a patient with chronic kidney disease (CKD) and a patient with AKI may present to the ED with a similar sCr. Therefore, accurate identification of AKI in an acute ED setting based upon sCr is challenging. Because to date AKI is defined by the level of creatinine, rather than biochemical outcomes and measures, consequently it is difficult to define what clinical parameters constitute a false positive elevated creatinine. As a result, creatinine is only useful as a retrospective biomarker of kidney function. In contrast biomarkers, such as NGAL, respond immediately to an injurious stimulus: they lack the delayed responses of serum creatinine. This is valuable for patients in the ED and triage decision-making. The potential to identify AKI both early and more accurately would greatly assist assignment of patients to proper levels of care and potentially improve clinical outcomes. In other words, earlier detection of AKI may permit timely interventions that may improve patient management and reduce costs.

The incidence of AKI in hospitalized patients ranges from 5–7% and is rising rapidly.[22–25] In a multinational study published in 2005 of critically ill patients, the prevalence of AKI requiring dialysis was 5.7% with a mortality rate of 60.3%.[26] From 2001 to 2011, data from US centers suggest that while the prevalence of dialysis-requiring acute kidney injury has increased, the mortality rate has decreased (from 28.0 to 19.7%).[27] In addition, patients with AKI have a higher risk for developing other non-renal co-morbidities [28] and when present in conjunction with other conditions, AKI is associated with higher mortality.[2, 29, 30] Although no treatment to reverse AKI exists, early detection assists pre-emptive assignment of patients to higher levels of care. In JACC 2012, the authors found that Cr+/NGAL- or Cr-/NGAL+ have a 5% chance of dialysis or death within 7 days.[9] In contrast, NGAL+/Cr+ had a risk of 15% in 7 days. Nearly identical data were obtained when KIM-1 was studied.[9] Hence, the authors demonstrated, the combination of biomarkers represents a higher risk state than either alone. Indeed, the high morbidity and mortality of AKI in part reflects poor early detection due to the lack of an acute kidney injury biomarker and delay in placement to a higher level of acuity care.

Our investigation suggests that when sCr is in a relatively non-specific range for the diagnosis of AKI, using a combination of uNGAL and sCr leads both to a clinical benefit and an economic savings over sCr alone. The use of uNGAL in the ED leads to better triage decisions and a $408 per patient and $522 per patient savings at NYP-Allen and SIUH, respectively. Although in populations where sCr has high diagnostic accuracy for AKI the additional use of uNGAL may not result in cost savings, it may lead to improved patient management and by inference, better clinical outcomes. Our sensitivity analysis further shows that the superiority of using uNGAL and sCr is contingent on the total hospital costs, length of stay, and costs of additional testing when AKI is suspected. Additionally, the ability to identify patients with CKD is another area where the addition of uNGAL is economically beneficial.

Despite growing evidence to support uNGAL as an AKI diagnostic test, there are no data regarding its economic impact. Our investigation provides the first cost analysis of uNGAL utilization in AKI detection. We developed a model that assumed that the “real world” clinical implementation of uNGAL would be in conjunction with sCr. Patient data to support this analysis was derived from patients who presented with suspected AKI to the EDs at two U.S. medical centers located in New York City that serve two very different communities: one an inner-city academic center and the other a community-based hospital. Although this model suggests that the addition of uNGAL to sCr for AKI detection may lead to higher up-front direct costs, these initial costs are offset by potential savings due to reductions in delayed diagnosis and treatment. These models demonstrate that uNGAL+sCr resulted in 1,578 and 1,973 fewer patients experiencing delayed diagnosis and treatment at Center 1 (AH-NYPH) and Center 2 (SIUH), respectively. These findings are not surprising given the poor specificity of sCr in the population of patients selected for this analysis.

Cost estimates in these analyses are likely “conservative.” We expect higher costs would result from treatment delays due to delayed diagnosis, leading to greater illness severity, and therefore poorer clinical outcome and higher levels of inpatient care.[31–34] Therefore, these results suggest that cost savings achieved by adding uNGAL to sCr are proportional to avoiding those additional costs incurred with delayed diagnosis. Treatment delays increase costs because patients improperly diagnosed as being AKI-negative return for repeat testing in addition to eventual treatment and inpatient admission. Although treating patients unnecessarily adds costs, we expect false positive diagnoses would have short hospital stays, mitigating cost increases due to unnecessary treatment. Hence in this analysis, errors of commission (e.g., treating patients unnecessarily) are less costly then errors of omission (e.g., inappropriate diagnoses and treatment delays). Likely patients will be admitted for other diagnoses for which for the sake of simplicity our model does not account. However, early recognition of AKI will lead to early treatment and lower costs.

This investigation has limitations. First, this is a simulation model rather than a prospective investigation. Typical for these models, data must be collected from a variety of sources, including published studies and cost reports; clinical judgment and assumptions are utilized in cases where the former is unavailable. Model inputs may have varying accuracy or relevance in different medical settings. The model also assumes that patients, initially who were false-negatives, on follow-up would be at the same AKI severity level as when they were initially evaluated. However, we choose study sites serving different socioeconomic groups with different prevalence rates of comorbidities in order to increase the generalizability of our models. Furthermore, the results are robust in sensitivity analysis where the underlying variables are tested throughout a wide range of values likely to be encountered in clinical practice; the cost advantage of uNGAL+sCr persists over a wide range of costs and clinical values. For example, although one source was used to estimate the cost of creatinine, multiple values were tested through the sensitivity analysis. The savings benefit of uNGAL+sCr relies upon the total hospital costs, length of stay, the costs of additional testing when AKI is suspected, and the ability to identify patients with CKD. Although these models do not account for acute dialysis, quality of life, and mortality, we would expect these poor clinical outcomes to increase cost, in particular for those patients inappropriately given a false negative AKI diagnosis.

This analysis of uNGAL as an AKI diagnostic test suggests that combining uNGAL with sCr results both in clinical and cost benefits in specific settings. When sCr is in a non-specific range for AKI detection, combining uNGAL with sCr may lead to earlier AKI detection and treatment and avoidance of an inappropriate false negative diagnosis of AKI. Per patient savings ranged from $408—$522 at two separate centers. Longitudinal studies are necessary to investigate the cost-effectiveness of uNGAL, used both with and without sCr, for the diagnosis of AKI.

## Appendix

### Additional methods: Evaluations of diagnostic test characteristics and assignment of gold standard test status

To evaluate each test’s diagnostic precision, we calculated their positive and negative predictive values (PPV and NPV, respectively). For this investigation, we assigned ‘gold standard’ test status to the uNGAL+sCr combination for both AKI detection and for decision to continue with further testing or send home (i.e. delaying treatment) as the combination provided the greatest amount of diagnostic information in comparison to either test alone. Therefore, delaying treatment was decided to be unambiguously appropriate if both uNGAL and sCr, adjusted for their false negative rate, were below pre-assigned cutoff levels. Comparisons were then made between uNGAL+sCr and sCr alone groups for delayed treatment rates. A similar approach was used to evaluate prevalence estimates of patients who were determined to have been treated unnecessarily. To determine if further testing was unambiguously required, we used the number of patients treated in the uNGAL+sCr arm, adjusted for the false positive rate, yielding the number of patients who unambiguously required further treatment. This was then compared to the number of patients treated in the sCr arm to determine how many patients were treated unnecessarily in that arm. In addition, the following model assumptions were made: 1) false negatives would become symptomatic for AKI and require further diagnostic evaluation; and 2) false positives underwent further AKI diagnostic evaluation.

Finally, we estimated the joint sensitivity (PPV) and specificity (NPV) for uNGAL+sCr by assuming the PPV and NPV of the combined measures were equivalent to the PPV of NPV of the highest measure. Therefore, if the PPVs of uNGAL and sCr were 0.5 and 0.4, respectively, the joint PPV was 0.5. This approach assumed that given the same PPV and NPV each test would result in the same individuals considered as being either truly positive or negative. This was done to provide the most conservative estimate of the joint probability. If we had assumed the PPV and NPV of each diagnostic test was independent, then an additive model would be required (e.g, 0.5 + 0.4 = 0.9), which might artificially inflate the joint probabilities. The decision trees describing the treatment patterns under sCr and uNGAL+sCr are shown in Fig 1A and 1B, respectively.

### Assignment of kidney function diagnosis

#### Adjudications

The patients’ clinical course was evaluated by investigators blinded to biomarker data, who assigned adjudications and hence diagnosis (ES, PAC, KSO, TLN). Estimated glomerular filtration rate (eGFR) was calculated using the Modification of Diet in Renal Disease (MDRD) formula.[35] Patients were assigned to one of four diagnoses based upon strictly defined criteria as detailed below^15^:

**Normal kidney function**: (1) Baseline eGFR >60 ml/min/1.73m^2^; (2) Failure to meet minimal RIFLE criteria for AKI;[18] (3) no transient or sustained changes in sCr during the first three days of hospitalization (≥0.3 mg/dl when baseline sCr was ≥1.0 mg/dl, or ≥0.2 mg/dl when the baseline sCr was ≤1 mg/dl); and (4) no recent exposures to AKI risk factors (shock requiring vasopressors, positive blood cultures, SIRS or sepsis, nephrolithiasis, recent chemotherapy, nephrotoxins, rhabdomyolysis, glomerulonephritis, acute interstitial nephritis) or dialysis therapy.
**Stable CKD**: Identical characteristics as patients with normal kidney function but baseline eGFR <60 ml/min/1.73m^2^ and stable >3 months prior to admission.
**Prerenal Azotemia**: Patients who met minimal RIFLE criteria for AKI (1.5-fold increase in sCr or a 25% decrease in eGFR from baseline) at admission, but normalized their values within three days after admission. Their historical and laboratory data suggested decreased kidney perfusion, rather than exposure to AKI risk factors. They typically were treated with measures to restore perfusion, such as fluids or discontinuation of diuretics.
**AKI**: These patients met minimal RIFLE criteria for AKI which failed to normalize within three days following admission despite fluid therapy. Any patient who had a compelling reason for AKI that occurred after urine sampling (for example a new radiocontrast study or overdose with a nephrotoxin) was excluded.

#### sCr and uNGAL measures

Investigators assaying sCr and uNGAL were blinded to the diagnostic adjudications (KF, MS). In addition uNGAL measurements were unavailable to physicians who were responsible for the clinical management of the patients enrolled in this investigation. uNGAL was considered elevated if its value exceeded 125 ng/dL and sCr was considered elevated if its value exceeded 1.75 mg/dL. In a sensitivity analysis (detailed later), alternative values of sCr were used to define lower bound estimates of elevated sCr (1.5 mg/dL) and upper bound estimates of elevated sCr (> 2 mg/dL). Likewise, alternative values of uNGAL were considered for lower bound (>100 ng/dL) and upper bound (> 150 ng/dL) estimates of elevated uNGAL. Based on these cutoff values, the prevalence of patients with elevated sCr and uNGAL values were obtained from the two sites.

## References

[1] Fischer MJ, Brimhall BB, Lezotte DC, Glazner JE, Parikh CR. Uncomplicated acute renal failure and hospital resource utilization: a retrospective multicenter analysis. Am J Kidney Dis. 2005;46(6):1049–57. Epub 2005/11/29. 10.1053/j.ajkd.2005.09.006. 16310570

[2] Chertow GM, Burdick E, Honour M, Bonventre JV, Bates DW. Acute kidney injury, mortality, length of stay, and costs in hospitalized patients. J Am Soc Nephrol. 2005;16(11):3365–70. Epub 2005/09/24. 10.1681/ASN.2004090740. 16177006

[3] Wagener G, Gubitosa G, Wang S, Borregaard N, Kim M, Lee HT. Urinary neutrophil gelatinase-associated lipocalin and acute kidney injury after cardiac surgery. Am J Kidney Dis. 2008;52(3):425–33. Epub 2008/07/25. 10.1053/j.ajkd.2008.05.018. 18649981

[4] Ling W, Zhaohui N, Ben H, Leyi G, Jianping L, Huili D, et al Urinary IL-18 and NGAL as early predictive biomarkers in contrast-induced nephropathy after coronary angiography. Nephron Clin Pract. 2008;108(3):c176–81. Epub 2008/02/22. 10.1159/000117814. 18287807

[5] Siew ED, Ware LB, Gebretsadik T, Shintani A, Moons KG, Wickersham N, et al Urine neutrophil gelatinase-associated lipocalin moderately predicts acute kidney injury in critically ill adults. J Am Soc Nephrol. 2009;20(8):1823–32. Epub 2009/07/25. 10.1681/ASN.2008070673. 19628673 PMC2723988

[6] Parikh CR, Jani A, Mishra J, Ma Q, Kelly C, Barasch J, et al Urine NGAL and IL-18 are predictive biomarkers for delayed graft function following kidney transplantation. Am J Transplant. 2006;6(7):1639–45. Epub 2006/07/11. 10.1111/j.1600-6143.2006.01352.x. 16827865

[7] Nickolas TL, O'Rourke MJ, Yang J, Sise ME, Canetta PA, Barasch N, et al Sensitivity and specificity of a single emergency department measurement of urinary neutrophil gelatinase-associated lipocalin for diagnosing acute kidney injury. Ann Intern Med. 2008;148(11):810–9. Epub 2008/06/04. doi: 148/11/810 [pii]. 18519927 10.7326/0003-4819-148-11-200806030-00003 PMC2909852

[8] Shapiro NI, Trzeciak S, Hollander JE, Birkhahn R, Otero R, Osborn TM, et al The diagnostic accuracy of plasma neutrophil gelatinase-associated lipocalin in the prediction of acute kidney injury in emergency department patients with suspected sepsis. Ann Emerg Med. 56(1):52–9 e1. Epub 2010/04/07. 10.1016/j.annemergmed.2010.02.010. 20363526

[9] Nickolas TL, Schmidt-Ott KM, Canetta P, Forster C, Singer E, Sise M, et al Diagnostic and prognostic stratification in the emergency department using urinary biomarkers of nephron damage: a multicenter prospective cohort study. Journal of the American College of Cardiology. 2012;59(3):246–55. 10.1016/j.jacc.2011.10.854. 22240130 PMC3487165

[10] Kate RJ, Perez RM, Mazumdar D, Pasupathy KS, Nilakantan V. Prediction and detection models for acute kidney injury in hospitalized older adults. BMC Med Inform Decis Mak. 2016;16:39 10.1186/s12911-016-0277-4. 27025458 PMC4812614

[11] Bedford M, Stevens P, Coulton S, Billings J, Farr M, Wheeler T, et al Development of risk models for the prediction of new or worsening acute kidney injury on or during hospital admission: a cohort and nested study Health Services and Delivery Research. Southampton (UK) 2016. 26937542

[12] Nickolas TL. Comparison of Urinary Biomarkers in the Diagnosis of Acute Kidney Injury and the Prediction of Morbidity and Mortality at Hospital Admission: An International Multicenter Study. In Review. 2010.

[13] Clinical Laboratory Fee Schedule: Centers for Medicare and Medicaid Services; 2010 [updated 08/29/201008/29/2010]. http://www.cms.gov/ClinicalLabFeeSched/02_clinlab.asp-TopOfPage.

[14] Zwang O, Albert RK. Analysis of strategies to improve cost effectiveness of blood cultures. J Hosp Med. 2006;1(5):272–6. Epub 2007/01/16. 10.1002/jhm.115. 17219512

[15] Pronovost PJ, Needham DM, Waters H, Birkmeyer CM, Calinawan JR, Birkmeyer JD, et al Intensive care unit physician staffing: financial modeling of the Leapfrog standard. Crit Care Med. 2006;34(3 Suppl):S18–24. Epub 2006/02/16. 10.1097/01.CCM.0000208369.12812.92. 16477199

[16] Bellomo R, Ronco C, Kellum JA, Mehta RL, Palevsky P. Acute renal failure—definition, outcome measures, animal models, fluid therapy and information technology needs: the Second International Consensus Conference of the Acute Dialysis Quality Initiative (ADQI) Group. Crit Care. 2004;8(4):R204–12. Epub 2004/08/18. 10.1186/cc2872. 15312219 PMC522841

[17] Mehta RL, Kellum JA, Shah SV, Molitoris BA, Ronco C, Warnock DG, et al Acute Kidney Injury Network: report of an initiative to improve outcomes in acute kidney injury. Crit Care. 2007;11(2):R31 Epub 2007/03/03. 10.1186/cc5713. 17331245 PMC2206446

[18] Bellomo R, Ronco C, Kellum JA, Mehta RL, Palevsky P. Acute renal failure—definition, outcome measures, animal models, fluid therapy and information technology needs: the Second International Consensus Conference of the Acute Dialysis Quality Initiative (ADQI) Group. Crit Care. 2004;8(4):R204–12. Epub 2004/08/18. 10.1186/cc2872. 15312219 PMC522841

[19] Uchino S, Bellomo R, Goldsmith D, Bates S, Ronco C. An assessment of the RIFLE criteria for acute renal failure in hospitalized patients. Crit Care Med. 2006;34(7):1913–7. Epub 2006/05/23. 10.1097/01.CCM.0000224227.70642.4F. 16715038

[20] Haase M, Bellomo R, Devarajan P, Schlattmann P, Haase-Fielitz A. Accuracy of neutrophil gelatinase-associated lipocalin (NGAL) in diagnosis and prognosis in acute kidney injury: a systematic review and meta-analysis. Am J Kidney Dis. 2009;54(6):1012–24. Epub 2009/10/24. 10.1053/j.ajkd.2009.07.020. 19850388

[21] Levey AS, Stevens LA, Schmid CH, Zhang YL, Castro AF 3rd, Feldman HI, et al A new equation to estimate glomerular filtration rate. Ann Intern Med. 2009;150(9):604–12. Epub 2009/05/06. doi: 150/9/604 [pii]. 19414839 10.7326/0003-4819-150-9-200905050-00006 PMC2763564

[22] Nickolas TL, Barasch J, Devarajan P. Biomarkers in acute and chronic kidney disease. Curr Opin Nephrol Hypertens. 2008;17(2):127–32. Epub 2008/02/16. 10.1097/MNH.0b013e3282f4e525. 18277143

[23] Hou SH, Bushinsky DA, Wish JB, Cohen JJ, Harrington JT. Hospital-acquired renal insufficiency: a prospective study. Am J Med. 1983;74(2):243–8. Epub 1983/02/01. 6824004 10.1016/0002-9343(83)90618-6

[24] Shusterman N, Strom BL, Murray TG, Morrison G, West SL, Maislin G. Risk factors and outcome of hospital-acquired acute renal failure. Clinical epidemiologic study. Am J Med. 1987;83(1):65–71. Epub 1987/07/01. doi: 0002-9343(87)90498-0 [pii]. 3605183 10.1016/0002-9343(87)90498-0

[25] Nash K, Hafeez A, Hou S. Hospital-acquired renal insufficiency. Am J Kidney Dis. 2002;39(5):930–6. Epub 2002/04/30. 10.1053/ajkd.2002.32766. 11979336

[26] Uchino S, Kellum JA, Bellomo R, Doig GS, Morimatsu H, Morgera S, et al Acute renal failure in critically ill patients: a multinational, multicenter study. JAMA. 2005;294(7):813–8. Epub 2005/08/18. 10.1001/jama.294.7.813. 16106006

[27] Brown JR, Rezaee ME, Hisey WM, Cox KC, Matheny ME, Sarnak MJ. Reduced Mortality Associated with Acute Kidney Injury Requiring Dialysis in the United States. Am J Nephrol. 2016;43(4):261–70. 10.1159/000445846. 27161485 PMC4899228

[28] Chertow GM, Soroko SH, Paganini EP, Cho KC, Himmelfarb J, Ikizler TA, et al Mortality after acute renal failure: models for prognostic stratification and risk adjustment. Kidney Int. 2006;70(6):1120–6. Epub 2006/07/20. 10.1038/sj.ki.5001579. 16850028

[29] Bellomo R. The epidemiology of acute renal failure: 1975 versus 2005. Curr Opin Crit Care. 2006;12(6):557–60. Epub 2006/11/02. 10.1097/01.ccx.0000247443.86628.68. 17077686

[30] Ympa YP, Sakr Y, Reinhart K, Vincent JL. Has mortality from acute renal failure decreased? A systematic review of the literature. Am J Med. 2005;118(8):827–32. Epub 2005/08/09. 10.1016/j.amjmed.2005.01.069. 16084171

[31] Wavamunno MD, Harris DC. The need for early nephrology referral. Kidney Int Suppl. 2005;(94):S128–32. Epub 2005/03/09. 10.1111/j.1523-1755.2005.09429.x. 15752229

[32] Obrador GT, Pereira BJ. Early referral to the nephrologist and timely initiation of renal replacement therapy: a paradigm shift in the management of patients with chronic renal failure. American journal of kidney diseases: the official journal of the National Kidney Foundation. 1998;31(3):398–417. Epub 1998/03/20. . 9506677 10.1053/ajkd.1998.v31.pm9506677

[33] Levin A. Consequences of late referral on patient outcomes. Nephrol Dial Transplant. 2000;15 Suppl 3:8–13. Epub 2000/10/14. . 11032351 10.1093/oxfordjournals.ndt.a027977

[34] Arora P, Obrador GT, Ruthazer R, Kausz AT, Meyer KB, Jenuleson CS, et al Prevalence, predictors, and consequences of late nephrology referral at a tertiary care center. Journal of the American Society of Nephrology: JASN. 1999;10(6):1281–6. Epub 1999/06/11. 10361866 10.1681/ASN.V1061281

[35] Levey AS, Bosch JP, Lewis JB, Greene T, Rogers N, Roth D. A more accurate method to estimate glomerular filtration rate from serum creatinine: a new prediction equation. Modification of Diet in Renal Disease Study Group. Ann Intern Med. 1999;130(6):461–70. Epub 1999/03/13. doi: 199903160-00002 [pii]. 10075613 10.7326/0003-4819-130-6-199903160-00002

