# Correction: Does NGAL reduce costs? A cost analysis of urine NGAL (uNGAL) & serum creatinine (sCr) for acute kidney injury (AKI) diagnosis

**DOI:** 10.1371/journal.pone.0185772

**Published:** 2017-09-27

**Authors:** Amay Parikh, John A. Rizzo, Pietro Canetta, Catherine Forster, Meghan Sise, Omar Maarouf, Eugenia Singer, Antje Elger, Saban Elitok, Kai Schmidt-Ott, Jonathan Barasch, Thomas L. Nickolas

The eleventh author’s name is spelled incorrectly. The correct name is: Jonathan Barasch.
